# Navigating international academic collaboration: The Arabic translation and cultural adaptation of the Quality Maternal and Newborn Care Framework index

**DOI:** 10.1371/journal.pone.0347114

**Published:** 2026-04-10

**Authors:** Khadeja Zaza, Andrew Symon, Sahar Hassan, Hadil Ali-Masri, Jenny McNeill, Janine Stockdale, Weam Alhulaibi, Berit Mortensen

**Affiliations:** 1 Faculty of Health Sciences, Oslo Metropolitan University, Oslo, Norway; 2 Mother and Infant Research Unit, School of Health Sciences, University of Dundee, Dundee, United Kingdom; 3 Department of Nursing and Master’s Program in Women’s Health, Faculty of Pharmacy, Nursing and Health Professions, Birzeit University, Birzeit, Palestine; 4 Women’s Health and Development Unit, Ministry of Health, Ramallah, Palestine; 5 School of Nursing and Midwifery, Queen’s University Belfast, Belfast, Northern Ireland; Mutah University, JORDAN

## Abstract

**Background:**

The Quality Maternal and Newborn Care Framework index (QMNCFi) is a validated English-language instrument developed to assess women’s experiences during pregnancy, childbirth, and the postnatal period. These experiences include respect, dignity, communication, continuity of care, and involvement in decision-making. Despite Arabic being spoken in 22 countries, substantial variations exist in dialects, terminology, and healthcare practices. Because maternity care models, communication styles, and clinical terminology vary across Arabic-speaking contexts, ensuring semantic and experiential equivalence is essential when adapting the QMNCFi for use in Middle Eastern settings. This study aimed to translate and culturally adapt the QMNCFi into Arabic and to document the international collaborative methodological process supporting this adaptation.

**Methods:**

We followed an internationally recognized six-stage approach for translation and cultural adaptation (forward translation, synthesis, back translation, expert committee review, pre-final testing, and finalization). Two research teams, representing institutions across five countries, collaborated under a unified protocol. Cognitive interviews were conducted with 48 postpartum participants in Palestine and the Kingdom of Saudi Arabia.

**Results:**

Refinements included adding a country identifier within demographic items to distinguish data collection sites, aligning demographic categories (e.g., education and income/currency), clarifying items with context-relevant examples, and adding “not applicable” where appropriate. These adjustments improved clarity and contextual fit without altering item order or scoring. Inter-rater agreement reached 99.1%, exceeding the a priori 80% benchmark. Operational challenges, including ethics approvals, data-sharing restrictions, supervision structures, and cross-site coordination, were addressed through structured mentorship, version-controlled documentation, and regular joint meetings.

**Conclusion:**

A shared language did not ensure semantic or experiential equivalence. Addressing institutional, regulatory, capacity, and linguistic factors was essential for developing a usable Arabic QMNCFi. This work provides a transparent methodological guideline for same-language, multi-country adaptation of health measurement tools and prepares the Arabic QMNCFi for psychometric validation and future implementation in Arabic-speaking maternity care settings.

## Introduction

Efforts to improve maternal and newborn health increasingly rely on standardized tools that capture women’s experiences, recognized by the World Health Organization (WHO) as essential to quality care [1]. These dimensions include respectful care, communication, autonomy, continuity of care, and access to information during pregnancy, childbirth, and the postnatal period. Adapting such instruments across languages and healthcare contexts requires careful methodological attention, as even validated indices require systematic translation, cultural adaptation, and pretesting to ensure semantic, conceptual, and experiential equivalence [2–6]. Experiential equivalence refers to the extent to which translated items accurately capture women’s lived experiences and reflect sociocultural realities within local healthcare systems. These challenges are amplified in regions where a shared language, such as Arabic, conceals significant variation in dialects, terminology, and social norms [5,7].

The Quality Maternal and Newborn Care (QMNC) Framework, published in the 2014 Lancet Series on Midwifery, identifies five domains of quality care: effective practices, organization of care, values, philosophy, and provider roles [8]. Based on this framework, the English-language 44-item Quality Maternal and Newborn Care Framework index (QMNCFi) was developed in 2023 through a Delphi consensus process to measure women’s experiences across antenatal, intrapartum, and postnatal care [9]. This version has demonstrated validity and reliability across diverse settings, including Australia, Ghana, India, and the United Kingdom [10].

While the QMNCFi has demonstrated cross-cultural utility, adapting it into Arabic presents distinct linguistic and contextual challenges**.** Arabic is spoken by over 400 million people across 22 countries and requires more than direct translation [11–13]. Modern Standard Arabic is primarily used in formal writing and is less common in everyday clinical communication [14]. Providers and women frequently switch between local dialects and English, particularly for clinical terminology [15,16]. For example, the term “blood pressure” may appear as ضغط الدم in formal Arabic, be shortened colloquially to “ضغط” in some Levantine contexts, or be communicated directly in English in Gulf settings [12,17]. These linguistic variations illustrate how regional dialects intersect with healthcare practices, professional training, and institutional norms, influencing how women interpret and respond to survey items.

Previous Arabic adaptations of health measurement instruments, including postpartum quality-of-life measures and chronic disease tools, have shown that shared language does not necessarily guarantee conceptual equivalence across settings [11,13]. These experiences highlight the importance of documenting adaptation decisions when implementing measurement instruments across Arabic-speaking populations. To address these challenges, researchers from Palestine, Norway, Scotland, the Kingdom of Saudi Arabia (KSA), and Northern Ireland established a multi-country collaboration. This study aimed to translate and culturally adapt the QMNCFi into Arabic using established cross-cultural adaptation guidelines and to provide a transparent methodological model for same-language, multi-country adaptation that may inform future global health measurement research.

## Materials and methods

### The index

The QMNCFi is a 44-item structured index developed to measure women’s perceptions of care quality during pregnancy, childbirth, and the postnatal period [8]. It covers five domains: effective practices, organization of care, values, philosophy, and provider roles [9]. Items use multiple response formats, including a 4-point Likert-type scale, with an additional “not applicable” option for specific items, binary responses (Yes/No), ternary responses (Yes/No/Not sure), and both single- and multiple-choice options. A brief socio-demographic section precedes the index to support contextualization and interpretation. Initially developed in English, the index has demonstrated construct validity and reliability in the United Kingdom, India, Ghana, and Australia in a single multi-country study [10]. To date, no Arabic version of the index has been implemented.

### Cross-cultural adaptation process

The cross-cultural adaptation of the QMNCFi into Arabic followed a six-stage process based on internationally recognized guidelines [4,5,18] (see S1 Fig in Supporting Information).

This approach incorporated both translation and cultural adaptation to achieve semantic, conceptual, and experiential equivalence: semantic (accurate translation of words and sentences), conceptual (shared meaning across contexts), and experiential (similar user experience). Two research teams from five countries collaborated using a unified protocol. Stages included forward translation, synthesis, back translation, expert committee review, pre-final testing, and finalization. Documentation was maintained using standardized templates and protocols established by the QMNC Research Alliance (www.qmnc.org) to ensure consistency.

Team 1 (Palestine, Norway, Scotland) led forward translation, synthesis, back translation, and expert review. Team 2 (KSA and Northern Ireland) contributed to pre-final testing and finalization. Methodological decisions regarding terminology, response options, and cultural adaptations were discussed in joint meetings until consensus was achieved.

#### Stage 1. Forward translation.

Two bilingual Arabic-English translators prepared independent versions. One translator with maternal health expertise prioritized clinical accuracy, while another translator emphasized participant-facing readability. Both documented translation challenges, terminology choices, and proposed adaptations.

#### Stage 2. Synthesis.

A maternal health expert reviewed both forward translations and synthesized them into a single Arabic version. The synthesis prioritized preservation of item intent, linguistic clarity, and cultural acceptability. All decisions were documented with explicit rationale in a synthesis report.

#### Stage 3. Back translation.

Two independent bilingual translators external to the project produced English back translations. They were certified in English proficiency and unfamiliar with the original index. This step aimed to detect semantic drift and confirm conceptual equivalence between the original English version and the synthesized Arabic version.

#### Stage 4. Expert committee review.

Before expert review, preliminary feedback was obtained from representatives of the target population, including two postpartum women, two midwives, and two obstetricians (one male and one female), all fluent in Arabic and English. They reviewed draft versions for clarity and cultural relevance.

A multidisciplinary panel then reviewed all (original English, forward translations, synthesized Arabic version, back translations, and user feedback). The panel included experts in psychometrics, maternal health, bilingual research, and cross-cultural adaptation.

Readability assessment was conducted during this stage to ensure participant suitability. The finalized wording achieved a Grade 6 reading level, indicating accessibility for women with varied educational backgrounds [4].

#### Stage 5. Testing pre-final version.

The pre-final Arabic index was tested with 48 postpartum women (28 in Palestine, 20 in KSA) using convenience sampling to assess clarity, comprehension, and usability. Convenience sampling was used because participants were recruited within routine clinical settings, allowing efficient cognitive testing during instrument refinement. The sample size aligns with cognitive interviewing recommendations, where approximately 30–50 participants are commonly sufficient to identify comprehension and contextual issues [10]. Recruitment and data collection occurred in February 2025. Eligible participants were Arabic-speaking women aged ≥18 years who gave birth between 6 and 52 weeks prior to participation and received care in at least one phase (antenatal, intrapartum, or postnatal) at a public facility [19,20].

Participant inclusion was aligned with the eligibility criteria applied in the original international QMNCFi validation study to ensure methodological comparability [10]. Participants received oral and written information, and informed consent was obtained orally and documented through an electronic “I agree” checkbox. Participants were assisted with reading when needed without prompts or suggested answers.

**Palestine site:** Recruitment occurred in vaccination clinics across the north, middle, and south of the West Bank. Participants completed the index in paper or online formats followed by remote cognitive interviews due to privacy and mobility considerations.
**KSA site:** Recruitment occurred face-to-face in a pediatric clinic within a large maternity hospital. Participants completed the index online using handheld devices followed by in-person cognitive interviews in a private setting.

Before interviews, site researchers reviewed completed indices for missing data, uniform response patterns, or inconsistencies. The cognitive interview guide included dichotomous clarity ratings (Clear/Unclear), word familiarity checks (Yes/No), and open-ended probes assessing meaning and relevance [21] (S1 File).

#### Stage 6. Finalization.

Reports summarizing participant feedback and response patterns were compiled and reviewed during structured joint research team meetings. Consensus was reached on all revisions to produce the final Arabic QMNCFi.

### Ethical considerations

This study involved the cross-cultural adaptation of a validated maternal health index with direct participation from postpartum women. All procedures adhered to the Declaration of Helsinki (2024 revision) [22]. No personal identifiers were collected, and data were de-identified prior to analysis.

Informed consent was obtained orally and documented through an electronic “I agree” checkbox. Participants were informed of their right to decline or withdraw without consequences.

Ethical approvals were obtained from Birzeit University (Ref: BZUPNH2139), the Palestinian Ministry of Health (Ref: 162/1255/2025), Queen’s University Belfast (Ref: MHLS 24_152), and the Institutional Review Board of the Maternity & Children’s Hospital in Al Ahsa (Ref: 390924-EP-2024).

Additional information regarding ethical, cultural, and scientific considerations related to inclusivity in global research is provided in the Supporting Information (S3 File).

## Results

### Translation and cross-cultural adaptation outcomes

Stages 1–4 (forward translation, synthesis, back translation, and expert committee review) were led by Team 1, while both teams contributed to testing and finalization. A small number of clinical terms, including “Primary caregiver,” “professional advice,” and “health facility,” lacked direct Arabic equivalents or varied by context. These were resolved through comparison of translations, expert review, and user feedback.

### Linguistic adaptations

A country-identifying item was added within the demographic section to allow distinction between data collection sites and alignment with national classifications. For example, Palestine and KSA required different income categories and currency formats. These changes did not alter the 44 core QMNCFi items.

### Contextual adaptations

In Stage 5, 48 postpartum women participated in cognitive interviews (28 in Palestine, 20 in KSA). Most items were understood as intended. Interview duration differed between sites, averaging approximately one hour in Palestine and approximately 15 minutes in KSA. This variation likely reflected differences in interview mode (remote versus face-to-face), participant engagement, and site logistics rather than item clarity, providing insight into operational differences across settings.

Women varied in age, education, delivery mode, and place of birth. Interviews identified wording issues requiring contextual adjustment. The inter-rater agreement reached 99.1%, calculated as percentage agreement between independent reviewer ratings, exceeding the predefined 80% benchmark recommended by Sousa and Rojjanasrirat (2011), indicating high clarity and cultural fit (S2 File).

### Cultural-sensitivity adaptations

Participant feedback led to targeted refinements. In Palestine, one participant considered the phrase “demonstrated good clinical skills” overly formal. The wording was revised to reflect everyday language while preserving the original meaning**.** Another participant reported that the family-respect item was not applicable because she gave birth alone, leading to the addition of a “not applicable” option without affecting scoring.

Five participants expressed discomfort with an alcohol-related information item, explaining that responses reflected cultural or religious norms rather than care experiences. The item was retained to preserve conceptual comparability, while a “not applicable” response option was introduced, illustrating how cultural sensitivity was balanced with framework consistency.

In KSA, participants requested clarification of educational materials. Examples such as “booklet” and “pamphlet” were added to improve comprehension. Routing questions about home-based care were also revised to better reflect mixed care pathways.

### Comparative synthesis between sites

Palestinian participants mainly highlighted language formality and contextual applicability, whereas KSA participants focused on clarification examples and routing logic. This comparison suggests that adaptation needs were driven more by healthcare system context and communication patterns than by language alone, reinforcing the importance of contextual adaptation even within shared-language settings.

All refinements were minor and maintained conceptual integrity and scoring structure. Refinements included adding a country identifier, aligning demographic categories, clarifying items with context-relevant examples, and adding “not applicable” where appropriate. These changes ensured that the index retained its meaning, improved clarity, and remained culturally relevant in both Palestine and KSA without compromising the original construct. Final revisions were reviewed and approved by both teams. The final Arabic version was then considered ready for psychometric validation.

### Operational insights and collaborative dynamics

The adaptation process followed established cross-cultural guidance. However, implementation was shaped by operational factors influencing workflow and decision-making.

Ethics approvals and data-sharing requirements differed across sites. Multi-layered approvals in Palestine and Norway contrasted with centralized processes in KSA. Restrictions on cross-border data transfer required sharing anonymized summaries rather than raw data, which preserved compliance but slowed reconciliation.

Differences in researcher experience and supervision structures also affected workflow. One researcher worked within a structured supervisory framework, while another relied more on peer support. These variations highlight how researcher capacity and institutional context can influence implementation consistency in multi-country adaptation studies.

Supervisors contributed complementary expertise in language, maternal health, and methodology. Digital collaboration required explicit documentation and version control to reduce ambiguity and prevent undocumented edits. Standardized reporting templates improved coordination in later stages.

Overall, operational findings indicate that successful same-language adaptation depends not only on linguistic expertise but also on coordinated governance, clear documentation, and collaborative infrastructure across sites.

## Discussion

This study translated and culturally adapted the QMNCFi into Arabic across two contexts, Palestine and KSA. Rather than repeating procedural results, this discussion provides a thematic synthesis showing how linguistic, contextual, and operational factors influenced equivalence during adaptation**.**

### Equivalence across contexts and the challenge of shared language

Although both settings share Arabic, the findings demonstrate that shared language did not guarantee experiential equivalence. Measurement validity depended on health system context, communication practices, and women’s expectations of care, consistent with equivalence models such as Herdman et al. [20], which distinguish semantic, conceptual, and experiential dimensions of cross-cultural adaptation.

Continuity-of-care items illustrate this challenge. In Palestine, interpretation occurred within a health system characterized by fragmented service delivery and movement restrictions, where continuity was often experienced as discontinuous or provider-dependent. In contrast, participants in KSA interpreted similar items within a more structured referral and postnatal education system. These differences suggest that experiential equivalence is shaped not only by translation accuracy but also by health system organization, clinical communication norms, and women’s expectations of care.

The findings therefore support the argument that linguistic equivalence alone is insufficient when adapting maternity care instruments across heterogeneous health systems. Adaptation must address how women interpret care through local institutional realities, levels of health literacy, and culturally shaped provider-patient interactions.

### Translation as an interpretive and negotiated process

Stages 1–4 highlighted that translation was not purely technical but interpretive. Terms lacking direct Arabic equivalents required negotiation between clinical precision and participant comprehension. This reinforces contemporary perspectives that translation represents an interpretive act rather than a literal transfer of wording.

The bilingual composition of the research teams enabled iterative mediation between formal language, spoken dialects, and methodological consistency. Researcher positionality therefore influenced decision-making, as translators and researchers acted simultaneously as linguistic mediators, cultural interpreters, and methodological gatekeepers.

Feedback during cognitive interviews further demonstrated that women interpreted items through personal, cultural, and healthcare experiences. The decision to retain culturally sensitive items (e.g., alcohol-related questions) while adding a “not applicable” option illustrates how adaptation required balancing cultural acceptability with conceptual fidelity to the original framework.

### Operational and collaborative influences on adaptation

Operational factors played a substantial role in shaping the adaptation process. Ethics approvals and data governance differed across sites, requiring anonymized data exchange rather than raw-data transfer. While this approach protected compliance, it slowed iterative reconciliation.

Variation in researcher training and supervision structures also affected workflow. These differences highlight that methodological rigor in cross-cultural adaptation depends not only on protocols but also on capacity building, mentorship, and collaboration structures.

Digital collaboration supported continuity but required version control and structured documentation to avoid inconsistencies. These findings suggest that successful multi-country adaptation is not only a linguistic task but also an organizational and governance process.

### Methodological and contextual limitations

Methodological limitations include reliance on convenience sampling and a modest qualitative sample size, although this aligns with established recommendations for cognitive interviewing in cross-cultural adaptation research.

Contextual limitations relate to conducting the study in only two Arabic-speaking settings. While Palestine and KSA represent distinct healthcare systems, findings may not capture the full linguistic and institutional diversity across Arab countries.

Translation decisions were achieved through expert consensus. Although consensus improves internal coherence, it also reflects interpretive judgments influenced by researcher perspectives, which should be acknowledged as part of the adaptation process rather than viewed solely as bias.

### Implications and future directions

The study demonstrates that adapting health measurement tools within the same language requires careful attention to experiential equivalence and institutional context. Future work should prioritize formal psychometric validation to confirm structural validity, reliability, and cross-country comparability of the Arabic QMNCFi.

Further testing across additional Arabic-speaking regions is needed to evaluate measurement

invariance and determine whether the instrument support regional benchmarking of maternity care experiences.

Beyond this instrument, the findings provide practical lessons for multi-country same-language adaptations, emphasizing early planning for ethics harmonization, shared documentation systems, and structured mentorship guidance.

### Synthesis

On completion of all stages, the Arabic QMNCFi achieved semantic, conceptual, and experiential equivalence sufficient for psychometric testing. The central insight of this study is that equivalence emerges through negotiation among language, health systems, and researcher mediation, rather than translation alone. These findings provide a practical foundation for future validation studies and support the use of standardized women-centered maternity care measurement tools across Arabic-speaking contexts.

## Conclusion

This study provides structured methodological guidance for same-language, multi-country adaptation. The Arabic QMNCFi is now prepared for psychometric validation and future implementation in Arabic-speaking maternity care settings. Beyond producing a linguistically accurate version, the study shows that adaptation extends beyond translation and requires systematic attention to experiential equivalence, health system context, and collaborative methodological design. The findings demonstrate that shared language does not guarantee shared interpretation, reinforcing the importance of integrating linguistic, institutional, and organizational factors during adaptation.

By documenting both technical and operational processes, this work offers reproducible guidance for cross-cultural adaptation within shared-language contexts and contributes methodological guidance to global health measurement research. These insights show how standardized instruments can be adapted while preserving conceptual integrity across culturally and institutionally diverse environments.

The final Arabic QMNCFi is positioned for psychometric validation. This will enable cross-country benchmarking of women’s maternity care experiences across Arabic-speaking contexts and support standardized quality-of-care monitoring at regional and international levels. Future validation will evaluate measurement invariance and strengthen the tool’s role in global maternal health evaluation frameworks.

## Supporting information

S1 Fig Arabic QMNCFi translation and cross-cultural adaptation process. (DOCX)

S1 File Cognitive debriefing guide. (DOCX)

S2 File Inter-rater agreement analysis. (DOCX)

S3 File Inclusivity in global research questionnaire. (DOCX)
