# Supplementary material for: Navigating international academic collaboration: The Arabic translation and cultural adaptation of the Quality Maternal and Newborn Care Framework index
Source: PLoS One. 2026 Apr 10;21(4):e0347114. doi: 10.1371/journal.pone.0347114 (PMC13068278; doi:10.1371/journal.pone.0347114)
Supplement: S1 Fig — (DOCX) [file pone.0347114.s001.docx]

**S1 Fig. Arabic QMNCFi translation and cross-cultural adaptation process.**

Workflow across six stages of translation and cross-cultural adaptation of the QMNCFi into Arabic.
