# Supplementary material for: Navigating international academic collaboration: The Arabic translation and cultural adaptation of the Quality Maternal and Newborn Care Framework index
Source: PLoS One. 2026 Apr 10;21(4):e0347114. doi: 10.1371/journal.pone.0347114 (PMC13068278; doi:10.1371/journal.pone.0347114)
Supplement: S1 File — (DOCX) [file pone.0347114.s002.docx]

**S1 File. Cognitive debriefing guide**

Form used during cognitive interviewing to assess participant understanding, clarity, wording familiarity, and interpretation of QMNCFi items during pre-testing (Stage 5).

**Example items from cognitive interviews**

| **Item** | **Question clarity (Clear / Not clear)** | **Response options clarity (Clear / Not clear)** | **Suggested change** | **Word familiarity (Yes / No)** | **Participant interpretation** |
| --- | --- | --- | --- | --- | --- |
| Item 1. My care provider(s) demonstrated good clinical skills | Not clear | Clear | Replace formal wording with simpler everyday Arabic | Yes | Participant understood this as whether the provider was competent and confident during care |
| Item 2. My care provider(s) respected my family members | Clear | Clear | Add “Not applicable” option for women who attended alone | Yes | Participant interpreted this as respectful behaviour toward accompanying relatives |
| Item 3. Were you offered information and/or relevant educational materials | Clear | Not clear | Add examples such as “booklet” or “pamphlet” | Yes | Participant understood it as receiving written explanations about care |
| Item 4. Were you offered information and/or relevant educational materials about alcohol | Clear | Clear | Keep item but add “Not applicable” due to cultural sensitivity | Yes | Participant interpreted the question as advice about avoiding alcohol during pregnancy |
| Item 5. Did you have all your planned care at home | Not clear | Not clear | Clarify wording about full versus partial home care and routing instruction | Yes | Participant interpreted this as whether every planned visit occurred at home or partly at a clinic |

*Source: Borsa et al., 2012 (1).*
