# Supplementary material for: Navigating international academic collaboration: The Arabic translation and cultural adaptation of the Quality Maternal and Newborn Care Framework index
Source: PLoS One. 2026 Apr 10;21(4):e0347114. doi: 10.1371/journal.pone.0347114 (PMC13068278; doi:10.1371/journal.pone.0347114)
Supplement: S2 File — (DOCX) [file pone.0347114.s003.docx]

**S2 File. Inter-rater agreement analysis**

Summary of items requiring revision during cross-cultural adaptation based on reviewer disagreement and participant feedback.

| **Section** | **Item No.** | **Item description** | **No. of disagreements (Country)** | **Comment** |
| --- | --- | --- | --- | --- |
| Part 1: Your care providers | 1.6 | My care provider(s) demonstrated good clinical skills | 1 (Palestine) | Wording felt too formal |
| Part 2: Your care providers’ values | 2.3 | My care providers respected my family members | 1 (Palestine) | Not applicable for women who gave birth alone; “Not applicable” option added |
| Part 3: The care you received | 3.8 | Were you offered information and/or relevant educational materials | 2 (KSA) | Participants requested examples such as “booklet” or “pamphlet” |
| Part 3: The care you received | 3.8f | Were you offered information and/or relevant educational materials about alcohol | 5 (Palestine) | Item considered culturally sensitive; “Not applicable” option added |
| Part 4: How your care was organized | 4.2 | Payment for care or insurance coverage | 1 (Palestine) | Clarification added to indicate financial costs |
| Part 4: How your care was organized | 4B | Did you have all your planned care at home? (If yes, skip to Q4.7) | 8 (KSA) | Skip pattern unclear; wording revised to distinguish complete vs partial home care |
| Part 5: Management of complications | — | No item requiring revision | 0 | None reported |

**Inter-rater agreement**

Inter-rater agreement was 99.1%, calculated as percent agreement between independent reviewers. This exceeded the predefined 80% benchmark.
