# Supplementary material for: Navigating international academic collaboration: The Arabic translation and cultural adaptation of the Quality Maternal and Newborn Care Framework index
Source: PLoS One. 2026 Apr 10;21(4):e0347114. doi: 10.1371/journal.pone.0347114 (PMC13068278; doi:10.1371/journal.pone.0347114)
Supplement: S3 File — (DOCX) [file pone.0347114.s004.docx]

Inclusivity in global research

PLOS’ policy on inclusivity in global research aims to improve transparency in the reporting of research performed outside of researchers’ own country or community and ensures that PLOS publications reporting global research adhere to high standards for research ethics and authorship .

This questionnaire outlines ethical, cultural, and scientific considerations relevant to inclusivity in global research. It is included as Supporting Information in accordance with journal requirements.

**Ethical considerations, permits and authorship**

Provide details as to who granted permissions and/or consent for the study to take place .

Ethical approvals were obtained from Birzeit University (Ref: BZUPNH2139), the Palestinian Ministry of Health (Ref: 162/250/2025), Queen’s University Belfast (Ref: MHLS 24_152), and the Institutional Review Board of the Maternity & Children’s Hospital in Al Ahsa (Ref: 390924-EP-2024). Full details are reported in the Methods section.

If there were any deviations from the study protocol after approval was obtained please provide details .

No deviations from the approved study protocol occurred after ethical approval was granted.

Did this study involve local collaborators that are residents of the country where the research was conducted or members of the community studied?

Yes. The study was conducted through an international collaboration involving researchers and institutions from five countries: Palestine, Norway, Scotland, the Kingdom of Saudi Arabia, and Northern Ireland. Data collection took place in two locations, Palestine and the Kingdom of Saudi Arabia. Local researchers in these countries were directly involved in study design, participant recruitment, data collection, interpretation of findings, and manuscript preparation.

**Authorship**

All authors meet PLOS authorship criteria and are appropriately listed in the author byline.

**Human subjects research (e.g. health research, medical research, cross-cultural psychology)**

Did you obtain written informed consent from a representative of the local community or region before the research took place?

This study did not require consent from a single community representative. Access and approval were obtained through institutional and governmental bodies responsible for healthcare delivery, including relevant ethics committees and health authorities in each setting. Informed consent was obtained directly from all participants and is described in the Methods section.

How did members of the local community provide input on the aims of the research investigation, its methodology, and its anticipated outcomes?

Postpartum women participated in cognitive interviews during the pre-testing stage and provided direct input on item clarity, cultural relevance, and interpretation. Their feedback informed revisions to wording and response options. In addition, midwives and obstetricians reviewed draft versions to ensure contextual and clinical appropriateness.

When engaging with the local community, how did you ensure that the informed consent documents and other materials could be understood by local stakeholders?

All materials were provided in Arabic. The instrument was translated and culturally adapted using established cross-cultural methods. Participants were offered oral explanations, and assistance with reading was provided when needed to ensure full understanding.

Will the findings of the research be made available in an understandable format to stakeholders in the community where the study was conducted (e.g. via a presentation, summary report, copies of publications, etc.)? Please provide details of how this will be achieved.

Yes. Findings will be shared with participants and community stakeholders in accessible formats. A plain-language summary will be developed to explain key findings and their implications. Community presentations or meetings will be offered. Printed and digital summaries will be distributed through local networks and partner institutions. Participants will be informed how to access results.

**Non-human subjects research using specimens/ animals collected as part of the study, or those housed in archival collections. Examples include archaeology, paleontology, botany and zoology.**

Did the permission you obtained from a local authority to perform the study include an agreement on access to outputs and benefit sharing?

Not applicable. This study involved human participants only and did not include animal specimens, archaeological materials, or biological sample collections.

If material was imported, provide details.

Not applicable. No materials were imported for this study.

If archival specimens were used, provide details.

Not applicable. This study did not involve archival specimens or previously collected materials.

How was cultural significance considered and were local researchers involved?

Not applicable. No physical materials or specimens were collected. The study involved human participants only.

If your manuscript includes photographs of human remains please indicate whether authors obtained permission from descendants or affiliated cultural communities to do so.

Not applicable. This study does not include photographs of human remains.
